# Factors associating with surgical site infection following operative management of malleolar fractures at an urban level 1 trauma center

**DOI:** 10.1097/OI9.0000000000000077

**Published:** 2020-05-06

**Authors:** Alexander S. Rascoe, Michael D. Kavanagh, Megan A. Audet, Emily Hu, Heather A. Vallier

**Affiliations:** MetroHealth Medical Center, Cleveland, Ohio, affiliated with Case Western Reserve University.

**Keywords:** ankle fracture, comorbidity, complication, infection, malleolar fracture, SSI, surgical site infection

## Abstract

**Objectives:**

To identify comorbidities and injury characteristics associated with surgical site infection (SSI) following internal fixation of malleolar fractures in an urban level 1 trauma setting.

**Design:**

Retrospective.

**Setting:**

Level 1 trauma center.

**Patients/Participants:**

Seven-hundred seventy-six consecutive patients with operatively managed malleolar fractures from 2006 to 2016.

**Intervention:**

Open reduction internal fixation.

**Main Outcome Measurements:**

Superficial SSI (erythema and drainage treated with oral antibiotics and wound care) or deep SSI (treated with surgical debridement and antibiotics).

**Results:**

Fifty-six (7.2%) patients developed SSI, with 17 (30%) of these being deep infections. An a-priori power analysis of n = 325 (α=0.05, β=0.2) was tabulated for differences in univariate analysis. Univariate analysis identified categorical associations (*P* < .05) between SSI and diabetes mellitus, drug abuse, open fracture, and renal disease but not tobacco abuse, body mass index, or neuropathy. Multivariate logistic regression identified categorical associations between diabetes (OR = 2.2, 95% CI: 1.1–4.3), drug abuse (OR = 3.9, 95% CI: 1.2–12.7), open fracture (OR = 4.1, 95% CI: 1.3–12.8), and renal disease (OR = 2.7, 95% CI: 1.4–5.0) and any (superficial or deep) SSI. A separate multivariate logistic regression analysis found categorical associations between deep SSI requiring reoperation and diabetes (OR = 4.4, 95% CI: 1.6–12.2) and open fracture (OR = 4.1, 95% CI: 1.3–12.8). Furthermore, American society of anesthesiologists classification (ASA) Class 4 patients were (OR = 9.2, 95% CI: 2.0–41.79) more likely to experience an SSI than ASA Class 1 patients.

**Conclusions:**

Factors associated with SSI following malleolar fracture surgery in a single urban level 1 trauma center included diabetes, drug abuse, renal disease, and open fracture. The presence of diabetes or open type fractures were associated with deep SSI requiring reoperation.

**Level of Evidence:**

Level 3 prognostic: retrospective cohort study.

## 1 Introduction

With an annual incidence of 187 per 100,000 person-years, ankle fractures are among the most commonly treated orthopaedic injuries and account for 9% of all fractures.^[1,2]^ Displaced torsional ankle fractures are treated with open reduction and internal fixation to provide better alignment and function.^[3–6]^ The risk of surgical site infection (SSI) following surgery is estimated between 1.44% and 14% and thought to be increased when treating patients with open fractures or diabetes mellitus.^[7–10]^ Other injury and patient characteristics including dislocation at time of injury, body mass index (BMI), peripheral neuropathy, renal disease, and ASA score may also correlate with the infection rate. Monetary costs of treating patients who develop SSI following malleolar fracture fixation are substantial, with a median total cost of $25,186 for patients who develop SSI versus $10,698 for those who do not.^[11]^

Given a paucity of data in the literature regarding SSI and ankle fractures from urban-based level 1 trauma centers, the present study was designed to elucidate patient injury characteristics and comorbidities that would associate with SSI. Scrupulous identification of factors that associate with SSI will inform operative treatment and postoperative management in this unique patient population; more informed surgeons can better advise and treat patients.

## 2 Patients and methods

We identified the hospital records of 982 skeletally mature patients with torsional ankle fractures treated surgically at an urban level 1 trauma center between 2006 and 2016. Patients were excluded if they were younger than 18 years old at the time of injury (n = 15) or were followed for fewer than 6 weeks from the time of injury (n = 191). This study was approved by the committee on research ethics at the institution in which the research was conducted in accordance with the Declaration of the World Medical Association (www.wma.net) and that any informed consent from human subjects was obtained as required. Following IRB approval, the electronic health record was queried for age, sex, race, BMI, cardiac arrhythmias, cancer history, history of cerebrovascular accident, diabetes, clinical neuropathy, psychiatric illness, pulmonary disease, renal disease, thromboembolic phenomena, tobacco use, alcohol abuse, illicit drug use, mechanism of injury, and ASA class. Fractures were classified according to the AO/OTA system.^[12]^ Injury characteristics including the presence of medial, lateral or posterior malleolar fractures, deltoid ligament injury, open fracture, or dislocation were recorded.

Based on the above criteria (n = 206 excluded), we identified 776 patients with mean age at the time of injury 45 years ± 16, and 382 (51%) were male (Table 1). Most patients were white (n = 479; 62%), and mean BMI was 31.1 kg/m^2^ ± 8.0. We determined that a sample size of n = 325 (α=0.05, β=0.2) would be necessary to establish differences between our univariate variables.

**Table 1:**
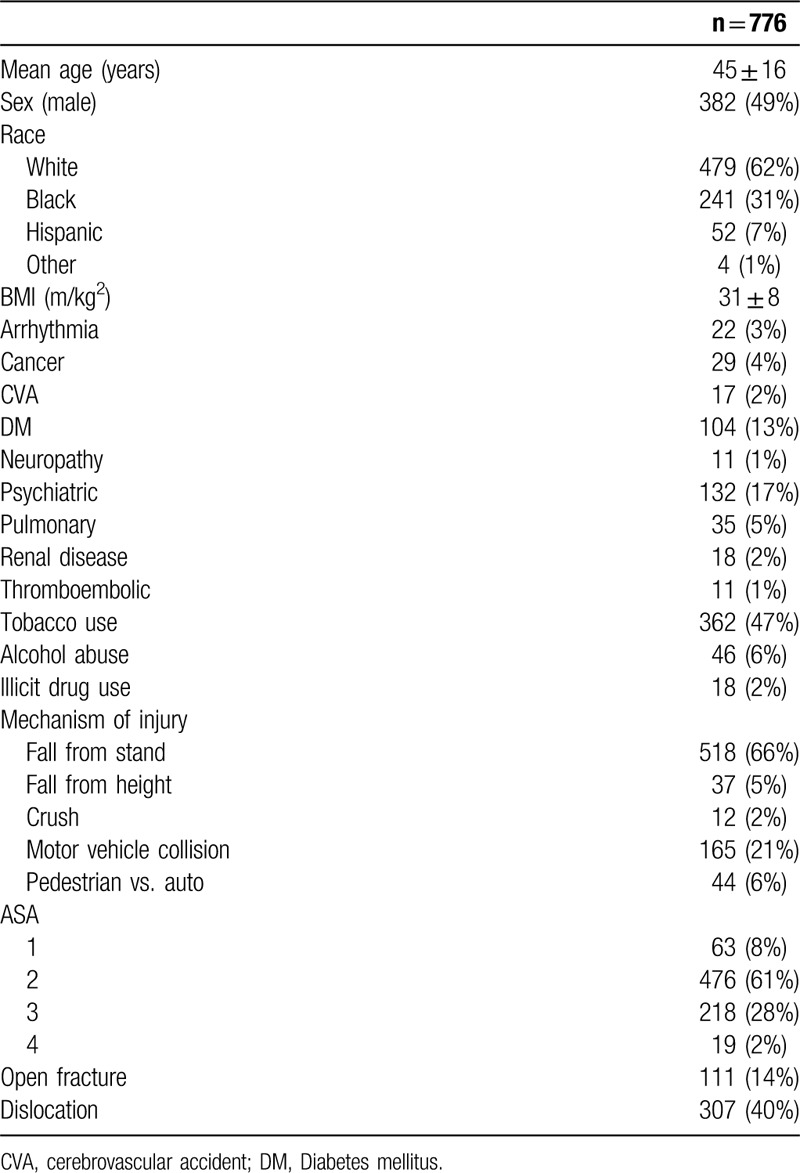
Demographic, injury, and social features of consecutive ankle fracture patients.

Patients were treated with conventional methods of reduction and fixation at surgeon discretion. Open fractures were treated with intravenous antibiotics at the time of presentation, and all underwent urgent surgical debridement followed by definitive fixation in the same setting or on a delayed basis at surgeon discretion, depending on injury and patient concerns. Perioperative antibiotics were standardized and included preoperative intravenous cephalosporin or equivalent, followed by one postoperative dose for patients treated on an outpatient basis and for 24 hours postoperatively for patients with open fractures.

Postoperative SSI was the primary variable and was defined as superficial: erythema and drainage treated with oral antibiotics and wound care, or deep: treated with surgical debridement and antibiotics.

Chi-squared analysis and logistic regression analysis were used for categorical variables and outcomes when applicable and ANOVA with Tukey's Honest Squared Different Post Hoc analysis for continuous variables when appropriate. ASA classes were compared by logistic regression analysis for OR (odds-ratio's) of associated SSI. Categorical variables identified as significant (*P* < .05) during initial univariate analysis were subsequently included in a multivariate logistic regression analysis for association with postoperative SSI and OR were calculated. Statistically significant factors were then analyzed with multivariate logistic regression for association with deep SSI requiring reoperation.

## 3 Results

From the patients identified, there were AO/OTA 44A (n = 6), 44B (n = 560), and 44C (n = 210) fractures, notably these fractures are defined as fibular fracture distal to syndesmosis, at the syndesmosis and proximal to the syndesmosis respectively. Mean time to surgery from date of injury was 7 days. Smoking was reported in 362 (47%), and most injuries resulted from ground level fall (n = 492; 63%). In total, 56 (7.2%) patients developed SSI with 39 superficial infections (5% of total, 70% of SSI) and 17 deep infections (2% of total, 30% of SSI) requiring return to the operating room for irrigation and debridement.

ASA class was associated with SSI, with ASA Class 4 patients more likely than Class 1 patients to experience infection with OR = 9.2 (95% CI: 2.0–41.79) (Table 2). The number of days from initial evaluation to definitive stabilization also significantly differed by ASA class (Table 3), with higher ASA class resulting in shorter time from initial evaluation to definitive stabilization.

**Table 2:**
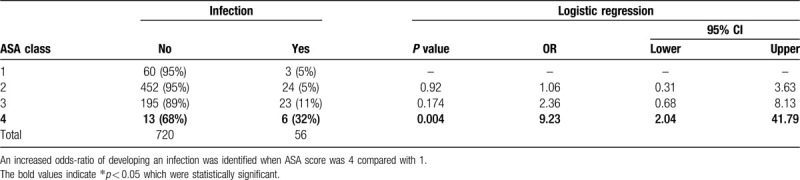
ASA classification and incidence of SSI (superficial or deep) following definitive fixation of operatively treated ankle fractures.

**Table 3:**
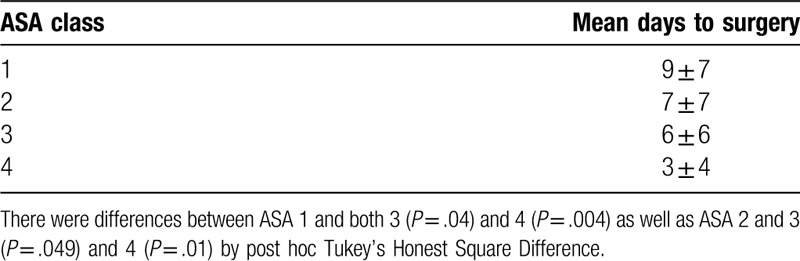
Mean number of days from initial evaluation to definitive stabilization stratified by ASA class.

Univariate analysis illustrated categorical associations between patient and injury characteristics and SSI. Patient comorbidities including diabetes (*P* = .02), presence of renal disease (*P* = .001), and illicit drug abuse (*P* = .001) were associated with postoperative SSI (Table 4). Further, open injuries (*P* < .001) were significantly associated with the development of SSI. Age greater than 65 years, clinical neuropathy, tobacco use or alcohol abuse, or dislocation were not associated with development of SSI.

**Table 4:**
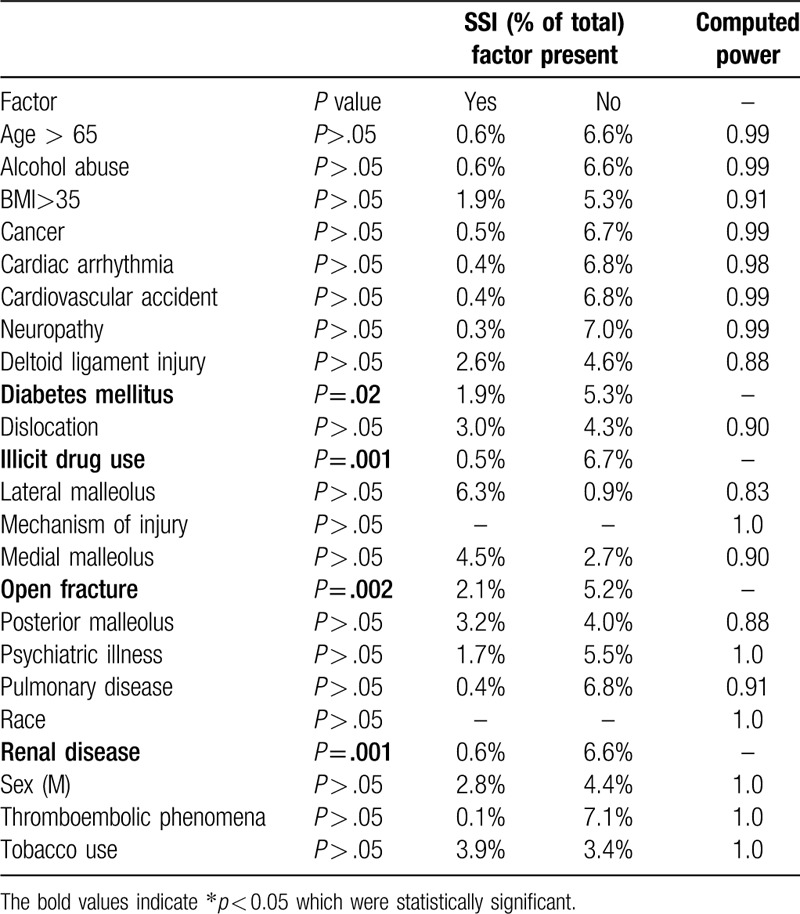
Chi-square analysis for categorical independent variables and their association with SSI (superficial or deep).

In a multivariate logistic regression analysis including factors that were statistically associated with infection in univariate analysis (Table 5), all factors tested were independently associated with SSI: diabetes (OR = 2.2, 95% CI: 1.1–4.3), drug abuse (OR = 3.9, 95% CI: 1.2–12.7), open fracture (OR = 4.1, 95% CI: 1.3–12.8), and renal disease (OR = 2.7, 95% CI: 1.4–5.0). These factors were then included in a multivariate logistic regression analysis for deep SSI requiring operation, and only diabetes (OR = 4.4, 95% CI: 1.6–12.2) and open fracture (OR = 4.1, 95% CI: 1.3–12.8) were significant. In patients with any 2 or more comorbidities or injury characteristics including diabetes, drug abuse, open fracture or renal disease, 10 (4.6%) developed a deep wound infection compared with 7 (1.3%) deep wound infections in patients who had no identified comorbidities or injury characteristics (*P* < .01).

**Table 5:**
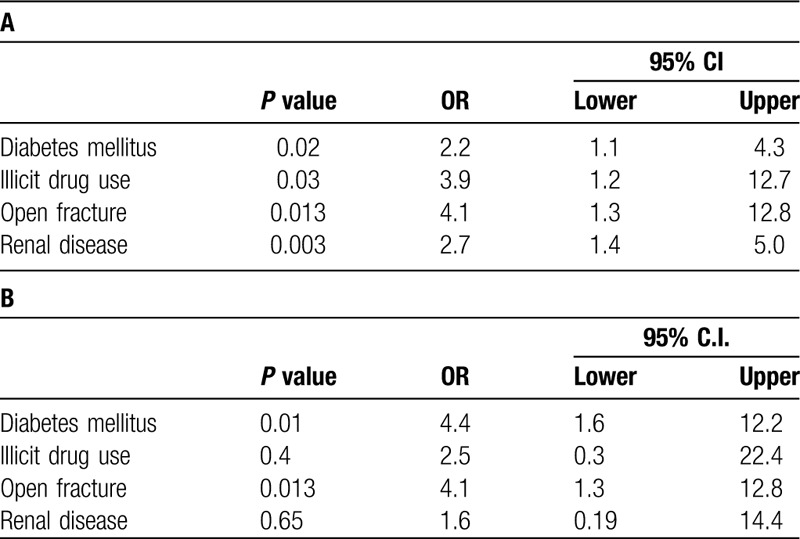
(A) Multivariate logistic regression analysis using statistically significant categorical variables identified in Chi-Square analysis (Table 4 for outcome of SSI (superficial or deep)); (B) multivariate logistic regression analysis for outcome of deep infection requiring repeat operation.

## 4 Discussion

We retrospectively reviewed more than a decade of experience in ankle fracture surgery at an urban level 1 trauma center. This work affirmed previous studies^[7,10]^ that associated both diabetes and open fracture with deep postoperative infections. An ASA Class 4 patient was (OR = 9.2, 95% CI: 2.0–41.79) more likely than an ASA Class 1 patient to have SSI in the study population (Table 2). This appears to represent a small but significant relationship between ASA Class 4 patients and SSI. Other groups have shown a similar relationship between increased ASA score and SSI following malleolar fracture surgery;^[8,13]^ however these studies included fewer ASA 3 patients, n = 47 and n = 87, and ASA Class 4 patients, both with n = 1. The relationship between ASA and length of stay in malleolar fracture surgery has been shown previously,^[14]^ and counter-intuitively, we found a relatively inverse relationship between ASA and number of days to definitive surgery. If higher ASA Class patients were admitted to the hospital from the emergency department to undergo medical optimization prior to surgery, this may explain this unexpected finding. Interestingly, a recent study has shown a higher Score for Trauma Triage in Geriatric and Middle Aged is predictive of both decreased likelihood of discharge home and longer mean length of stay.^[15]^

Two additional factors, renal disease and drug abuse, were associated with SSI in our urban patient population. Previous studies have associated total hip arthroplasty periprosthetic joint infection in patients with renal disease;^[16]^ this is the first study, to our knowledge, to associate renal disease and SSI following malleolar fracture surgery. Renal disease and its association with SSI may be confounded by the presence of diabetes. The present study design did not explicitly account for this, except for the use of a multivariate logistic regression analysis. Future studies should evaluate this potential confounding variable for SSI and carefully tease out the clinically important HbA1C levels in diabetics and GFR levels or dialysis use in patients with renal disease that associate with SSI.

Additionally, previous work has associated illicit drugs with a need for calcaneal screw removal following calcaneus fracture fixation^[17]^ and a positive urine drug screen associated with deep infection after malleolar fracture surgery.^[18]^ Our work provides additional evidence that self-reported illicit drug use in this urban population was associated with SSI following malleolar fracture surgery. In our urban-based population, where there is a high rate of intravenous drug abuse, further study would be needed to determine if intravenous drug abuse specifically or any illicit drug abuse, as an indirect measure of hygiene or self-care, correlates with SSI.

Surprisingly, no association between tobacco use and SSI was seen in our patient population, despite a high frequency of tobacco use within the studied population. Other studies have made similar conclusions,^[13,19]^ whereas a recent systematic review and other studies have identified associations between smoking and SSI.^[8,20,21]^ Tobacco smoking remains an important modifiable surgical risk factor, cessation of which reduces complication risks following fracture surgery,^[22]^ and although not supported by the results of this study, based on other available data, we do continue to recommend smoking cessation.

The current study identifies several associations for SSI following malleolar fracture surgery in an urban population. The association of ASA Class 4 and SSI (Table 2) weakly implies an association between increased comorbidities and SSI. While outpatient treatment would be more cost-effective, we would recommend ASA Class 4 patients have their surgeries performed in facilities and by physicians capable of managing the potential complications, both medically and surgically.^[23–26]^ As inappropriate transfers for higher level care occur at a rate of 16% to 52%^[27–29]^ and Bundled Payments for Care Improvement threatens to limit payments, we would encourage orthopaedic surgeons to advocate for higher levels of reimbursement for managing medically complex patients or as an orthopaedic community, carefully consider the value of nonoperative care in these patients.^[3–5]^

Our study evaluated ASA class, injury, and individual patient conditions associated with infection following malleolar fracture surgery in an urban population; we posit other perioperative fracture complications will also associate with patient comorbidities, which reflect host constitution. We found that in patients with any 2 comorbidities or injury characteristics including diabetes, drug abuse, open fracture or renal disease, 4.6% required return to the operating room for a deep wound infection compared with 1.3% of patients without these diagnoses. Surgeons evaluating patients with these conditions, or an ASA Class of 4 and a malleolar ankle fracture should carefully review these factors and SSI rates during the informed consent procedure. Further studies to evaluate patient comorbidities, injury patterns, clinical outcomes, and overall costs of care for operative versus nonoperative treatment of malleolar fractures in patients with severe comorbidity and SSI risk appear worthwhile.

There are several limitations to our study including the retrospective nature of data collection, which was constrained to documentation visible within the medical record, potentially leading to incomplete information. We believe rates of alcohol and tobacco consumption may be under appreciated in our study. Although our sample size is large (n = 776), only those patients who returned to our institution were included, which may have underestimated the true rate of SSI.^[30]^ Additionally as 20% of the initial sample (n = 206) patients were excluded from the review, their SSI rate remains unknown and if different from the remainder of the sample, may have altered the results.

Overall this study reviewed over a decade in the care of operative malleolar fractures at a single urban level 1 trauma center and evaluated the patient and injury characteristics which associate with perioperative risk for infection. Higher ASA class, open fractures, and diabetes were associated with SSI. Other comorbidities, such as renal disease and illicit drug use, also associated with infection in our patient population. In short, this study provides useful information regarding infection rates for the treatment of malleolar ankle fractures in an urban level 1 trauma setting.

## Acknowledgments

The authors gratefully acknowledge Dr Chang-Yeon Kim for his contributions with data refinement and analysis.
